# Prospective Zinc Solubilising Bacteria for Enhanced Nutrient Uptake and Growth Promotion in Maize (*Zea mays* L.)

**DOI:** 10.1155/2013/869697

**Published:** 2013-12-30

**Authors:** Praveen Kumar Goteti, Leo Daniel Amalraj Emmanuel, Suseelendra Desai, Mir Hassan Ahmed Shaik

**Affiliations:** Central Research Institute for Dryland Agriculture, Santoshnagar, Saidabad Post, Hyderabad, Andhra Pradesh 500 059, India

## Abstract

Zinc (Zn) is one of the essential micronutrients required for optimum plant growth. Substantial quantity of applied inorganic zinc in soil is converted into unavailable form. Zinc solubilising bacteria are potential alternates for zinc supplement. Among 10 strains screened for Zn solubilisation, P29, P33, and B40 produced 22.0 mm clear haloes on solid medium amended with ZnCO_3_. Similarly, P17 and B40 showed 31.0 mm zone in ZnO incorporated medium. P29 and B40 showed significant release of Zn in broth amended with ZnCO_3_ (17 and 16.8 ppm) and ZnO (18 and 17 ppm), respectively. The pH of the broth was almost acidic in all the cases ranging from 3.9 to 6.1 in ZnCO_3_ and from 4.1 to 6.4 in ZnO added medium. Short term pot culture experiment with maize revealed that seed bacterization with P29 @ 10 g*·*kg^−1^ significantly enhanced total dry mass (12.96 g) and uptake of N (2.268%), K (2.0%), Mn (60 ppm), and Zn (278.8 ppm).

## 1. Introduction

Zinc is one of the imperative micronutrients required relatively in small concentrations (5–100 mg kg^−1^) in tissues for healthy growth and reproduction of plants. Zinc deficiency in plants leads to reduced membrane integrity and synthesis of carbohydrates, auxins, nucleotides, cytochromes, and chlorophyll and develops susceptibility to heat stress [1]. Excessive use of zinc fertilizers also poses problems to humans causing the impaired absorption of iron and copper. It is also known to repress male sexuality [2]. The solubility of Zn is highly dependent upon soil pH and moisture and hence arid and semiarid areas of Indian agroecosystems are often zinc-deficient.

In India, maize is grown in a wide range of environments, extending from extreme semiarid to subhumid and humid regions. It is grown in about 8.26 Mha with yield being 19.3 Mt (Ministry of Agriculture, Government of India). Voluminous literature indicates that Zn concentration in the grain is inherently very low, particularly when grown on Zn-deficient soils. The major reason for the widespread occurrence of Zn deficiency problems in crop plants is attributed to low solubility of Zn in soils rather than a low total amount of Zn [3]. Customary application of inorganic zinc partially caters the plant need as 96–99% of applied Zn is converted into different insoluble forms depending upon the soil types and physicochemical reactions within 7 days of application [4]. Microbes are potential alternate that could cater plant zinc requirement by solubilising the complex zinc in soil. Several genera of rhizobacteria belonging to *Pseudomonas* spp. and *Bacillus* spp. are reported to solubilise zinc. Microbes solubilise the metal forms by protons, chelated ligands, and oxidoreductive systems present on the cell surface and membranes [5–7]. These bacteria also exhibit other traits beneficial to plants, such as production of phytohormones, antibiotics, siderophores, vitamins, antifungal substances, and hydrogen cyanide [8]. In this study we reported the *in vitro* zinc solubilisation ability of selected strains and their ability to enhance the growth of *Zea mays* L.

## 2. Materials and Methods

### 2.1. Bacterial Strains and Culture Conditions

Bacterial strains used in this study were obtained from the culture bank of Central Research Institute for Dryland Agriculture, Hyderabad, India. Five each of *Pseudomonas* spp. were designated as P17, P21, P29, P33, and P74 and *Bacillus* spp. as B40, B61, B114, B116, and B118. The cultures were originated from composite and rhizospheric soils of diverse rainfed agroecosystems of India. *Pseudomonas* and *Bacillus* spp. were maintained on King's B and nutrient agar medium at 4°C.

### 2.2. *In Vitro* Zinc Solubilization Assay

All the isolates were inoculated into liquid mineral salts medium (g·lit^−1^) specified by Saravanan et al. [9] containing dextrose: 10.0; (NH_4_)_2_SO_4_: 1.0; KCl: 0.2; K_2_HPO_4_: 0.1; MgSO_4_: 0.2; pH: 7.0 and insoluble Zn compound (ZnO and ZnCO_3_: 0.1%; Agar: 15.0 g) and autoclaved at 121°C for 20 min. Actively growing cultures of each strain were spot-inoculated (3 *µ*L) onto the agar and plates were incubated at 28°C for 48 h. The clearing zone around colony was recorded. Quantitative study of zinc solubilization was studied in 150 mL conical flasks containing 50 mL of liquid mineral salt medium. The broth was inoculated with 10 *µ*L of overnight grown bacterial inoculum and incubated for 72 h at 160 rpm in an incubator shaker at 28 ± 2°C. After incubation, the culture broth was centrifuged and the concentration of Zn in the supernatant was estimated in atomic absorption spectrophotometer (GBC, Australia).

### 2.3. Seed Bacterization

Maize seeds of cultivable variety were surface sterilized with 1% sodium hypochlorite for 5 min and washed five times with sterile distilled water. Seeds were treated with talc-based inoculum containing 10^8^ cfu·g^−1^ of each strain with 0.5% carboxymethylcellulose (CMC) as adhesive.

### 2.4. Pot Trial

The pot culture experiment was conducted in 10 kg plastic pots (20 cm dia) filled with 9 kg sterile red soil (presterilized for three consecutive days) with six replications for each treatment. Maize cultivable variety seeds treated with bacterial inoculant were sown and the glasshouse condition was set at 28 ± 2°C and 70% humidity. Pots were watered once in two days with sterile distilled water until 60 days. The experimental setup consisted of 15 treatments namely, five treatments each of *Bacillus* and *Pseudomonas* strains as seed dresser @ 10 mg·kg^−1^ seed (T1 to 10), commercially available zinc solubilizing bacteria (T11), farm yard manure (FYM) @ 10 kg·acre^−1^ (T12), seeds primed by soaking overnight in 1.0% ZnSO_4_ (T13), positive control @ ZnSO_4_ @ 10 kg·acre^−1^ (T14), and uninoculated control (T15).

### 2.5. Plant Growth Measurement

After 60 days of sowing (DAS), plants were uprooted from the pots carefully and biometric parameters like root volume, shoot length, leaf area (measured by LI 3100, Lincoln, Nebraska, USA leaf area meter), and dry mass of plants were recorded as the indicative of plant growth.

### 2.6. Nutrient Analyses

Dried plants were finely ground in a mortar and pestle to amorphous powder and 100 mg was taken in 150 mL conical flask containing 10 mL nitric acid (HNO_3_) and perchloric acid (HClO_4_) in 9 : 4 ratio. The flasks were placed on a hot plate and digested at 300°C until the entire plant material turned colourless. The extract was taken in 100 mL volumetric flask and the volume was made to 100 mL with distilled water. These samples were used for estimation of sodium, potassium, and calcium by flame photometer. Phosphorus was quantified by sulphomolybdic acid method [10]. Total nitrogen content of the plants was estimated by micro-Kjeldahl method [11]. Similarly, micronutrients such as iron, copper, manganese, zinc, and magnesium were estimated by atomic absorption spectrophotometer.

### 2.7. Statistical Analysis

The values presented are the means of two independent experiments each with six replicates performed at different occasions. Data obtained from all the experiments were subjected to two-way analysis of variance (ANOVA). Mean values between treatments were compared with Fisher's least significant difference (LSD) test (*P* < 0.05).

## 3. Results

### 3.1. Zinc Solubilization Activity

**Figure 1:**
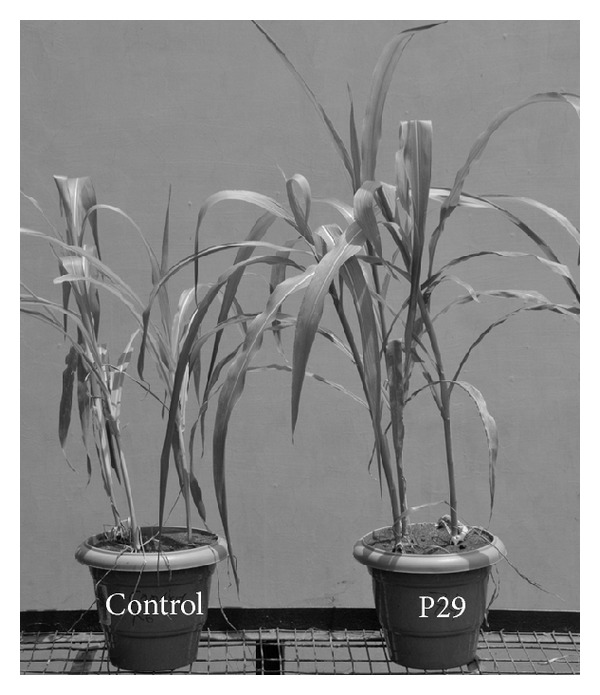
Plant growth promotion of maize with zinc solubilizing *Pseudomonas* sp. strain-P29 (60 DAS).

**Figure 2:**
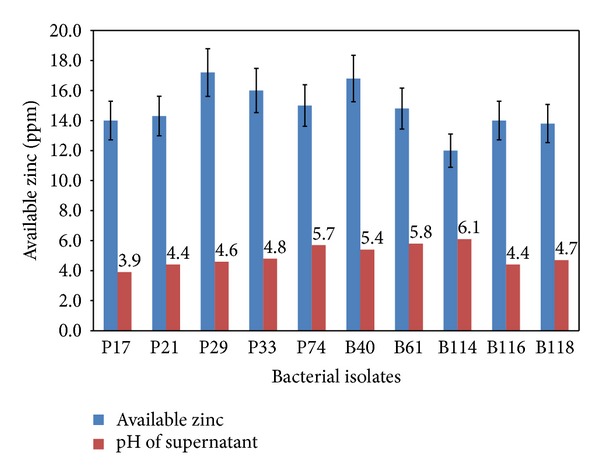
Available zinc (mg·kg^−1^) released by bacteria in broth medium containing zinc carbonate.

All the selected strains of *Pseudomonas* and *Bacillus* used could effectively solubilize the insoluble Zn compounds used, namely, ZnCO_3_ and ZnO, under the assay conditions. The zone of solubilisation was comparatively high in ZnO amended medium as compared to ZnCO_3_. Size of the solubilisation zone ranged from 14 to 22 mm in ZnCO_3_ and from 17 to 33 mm in ZnO incorporated medium. Among the cultures, P29, P33, and B40 showed the highest solubilisation zone in ZnCO_3_ (22 mm), whereas P17 and B40 showed 31 mm zone in ZnO amended medium (Table 1). Quantitative assay for zinc solubilisation revealed that P29, P33, and B40 were able to dissolve 17, 16, and 16.8 ppm from ZnCO_3_, respectively, in liquid medium (Figure 1) and they were consistent with the observations on solid medium. However, P17 which was found to be the leading solubilizer on plate agar did not imitate the result in broth amended with ZnO though significant fall in pH (4.1) was noted. Instead, P29 showed the highest Zn solubilisation of 18 ppm available Zn, followed by B40 (17 ppm) (Figure 2). Across the treatments, significant reduction of pH was observed in the broth cultures amended with ZnCO_3_ (pH 3.9–6.1) and ZnO (pH 4.1–6.4). However there was no significant correlation between the pH and solubilisation of nutrients.

### 3.2. Plant Growth Promoting Activity of Bacterial Strains

Seed bacterization of maize with zinc solubilising *Pseudomonas* spp. and *Bacillus* spp. enhanced the plant growth significantly after 60 DAS (Table 2). Among all the treatments, inoculation of maize with talc-based P29 strain showed increased root volume (18.3 cm^3^). Other growth indicating parameters like total dry mass (TDM) and leaf area (LA) were recorded to be the highest in ZnSO_4_ treatment with 15.25 g and 1161.3 cm^2^, respectively. However, they are statistically on par with P29 treatment where 12.96 g and 1147.5 cm^2^ of TDM and LA were recorded, respectively (Figure 1 and Table 2).

### 3.3. Nutrient Concentrations in Plants

Significant concentration of N (2.268%) and K (2.0%) was observed in P29 treatment. The highest P (0.28%) concentration was noted in B40 treated plants as compared to other treatments (Table 3). The highest sodium (Na^+^) concentration in plants was recorded in Zn primed, P29, P33, and ZnSO_4_ treatments which were not significantly different. Significant Ca (0.434%) concentration in plant tissues was found in ZnSO_4_ treatment followed by P74 (0.354%) and P29 (0.341%). Na–K ratio was higher with ZSB (0.0047) and priming (0.004) treatment; P29 (0.0033) and B118 (0.0029) also recorded the highest ratio of Na–K ions.

P29 significantly enhanced the concentration of Zn content (278.8 ppm) in plant tissue as high as 36, 32.35, and 43.11% compared to Zn priming, ZnSO_4_, and control treatment, respectively (Table 4). The highest Mn (60 ppm) concentration was also noted in P29 treatment which is on par with ZnSO_4_ treatments. Interestingly, P21 treated plants showed the highest quantity of Fe (707 ppm) which is statistically on par with B40 treatment (672.8 ppm). Mg (0.23 ppm) and Cu (4.8 ppm) were significantly found higher in inorganic ZnSO_4_ plants.

## 4. Discussion

Solubilisation of zinc can be accomplished by a range of mechanisms, which include excretion of metabolites such as organic acids, proton extrusion, or production of chelating agents [12, 13]. In addition, production of inorganic acids such as sulphuric acid, nitric acid, and carbonic acid could also facilitate the solubilisation [8, 14].

**Figure 3:**
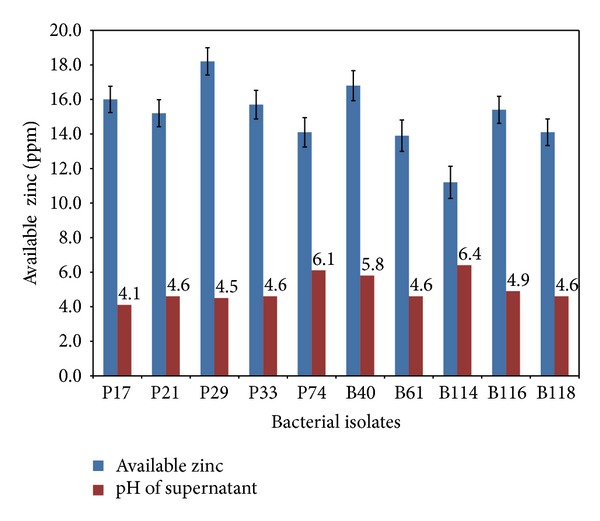
Available zinc (mg·kg^−1^) released by bacteria in broth medium containing zinc oxide.

It is apparent from the zinc solubilization data that the solubilization potential varied with each isolate. Organic acid production by microbial isolates has been reported to be a major mechanism of solubilization [15, 16]. This solubilization property is important in nutrient cycling. Fall in pH and acidification of medium was noted in all cases. Higher solubilization of the insoluble zinc sources was achieved in 72 h. The zinc solubilizing potential also correlated with the zinc levels that are accumulated by plant leaves. The zinc solubilization in our studies could be due to production of organic acids, like gluconic acids (especially 2-keto-gluconic acids). Zinc phosphate solubilization by a strain of *Pseudomonas fluorescens* was investigated by Di Simine et al. [17]. They identified that gluconic acids produced in culture medium helped in solubilization of zinc salts. In our present study also, the acidic pH shown by all the bacterial isolates gives a clue that the solubilization could be due to production of organic acids and higher the production of the same higher is the available zinc in the culture broth (Figure 3). Desai et al. [18] reported that higher availability of Zn is directly proportional to acidic pH of the culture broth. However, in some potent strains, pH did not fall drastically suggesting that in those strains other mechanisms may be active and this aspect is being accentuated.

The present study clearly demonstrated that inoculation with plant growth promoting rhizobacteria significantly enhanced the growth of maize in all dimensions. In this study application of ZnSO_4_ alone has increased the total dry mass of plants and leaf area. Increased leaf area with ZnSO_4_ alone was much similar to that of P29 treatment. P21 and P29 treated plants significantly enhanced the root volume and dry mass of plants, also supported by the studies carried out by Richardson [19], who showed that PGPR inoculation effectively increases surface area of roots and root weight [20]. The variation in enhancement of root volume by these strains may be due to the difference in the quantity of phytohormones produced by each strain. Auxin is a class of plant hormones of which indole-3-acetic acid is well studied, which has the capacity to enhance long term responses in plants [21]. *Pseudomonas* strains have increased root and shoot elongation in canola, lettuce, and tomato [22].

The plant growth promoting effects of bioinoculant PGPR strains were clearly demonstrated in many studies [23, 24]. The positive effects of PGPRs on yield and growth of maize were explained by Egamberdiyeva [25], which may be due to N_2_ fixation ability, P-solubilizing capacity, and phytohormones production.

From the tabulated data in Tables 2–4 it could be observed that no particular treatment with the bacterial strain or ZnSO_4_ or priming or FYM treatment could be able to enhance the growth of plants in all aspects. In case of dry mass and leaf area which were higher with the treatments where no bacteria were applied, much variation could not be seen on an overall comparison of treatments. This minor variation in between bacterial treatments could be due to difference in mechanisms of plant growth promotion exerted by different bacterial strains. Untreated plants showed poor growth.

In the current study, seed bacterization with zinc solubilizing plant growth promoting bacteria resulted in increased plant height (root volume and shoot height); leaf area; and dry mass. Similar increases in plant parameters were observed in different crops inoculated with *Pseudomonas*, *Azospirillum*, and *Azotobacter* strains [26, 27]. This improved growth by PGPR is due to making the increased availability of nutrients and decreasing metal toxicity [28].

The present study indicated that bacterial inoculation of maize with *Pseudomonas* and *Bacillus* significantly increased the nutrient content of “N” and “P” in leaves of maize (Table 3). This higher uptake of essential nutrients compared to uninoculated control plants could be justified from the fact that the unavailable forms of these nutrients were solubilized and made available in the root region by applying PGPR. Plants which are inoculated with plant growth promoting rhizobacteria usually have higher “N” content than that of uninoculated plants [29]. This fact is further strengthened by studies conducted by Murty and Ladha [30] who demonstrated that *Azospirillum* inoculation increased phosphate and ammonium uptake in rice plants. Even though K^+^ concentration was higher in other treatments than control, similar trend was not seen in case of Na^+^ levels (Table 3).

Results showed that all bacterial treated plants showed significant differences in Fe, Cu, Mn, and Zn content in maize leaves (Table 4) although differences between various bacterial strains were insignificant. The enhancement of macro- and micronutrient uptake by plants by inoculation with PGPR may be due to their effect on initiation and development of lateral roots [31], increased root weight, and nutrient uptake [32]. Studies by Goldstein and Liu [33] showed that phosphate and potash solubilizing bacteria may enhance mineral uptake in plants. This evidence confirmed that the percentage of various nutrients estimated in maize plants was significantly and/or relatively increased in bacterial treated plants (Table 4). On observing the critical Zn levels of treatments it can be said that the ZnSO_4_ treatment recorded lower Zn levels compared to other treatments. This can be explained from the fact that the presence of readily available zinc source in soil itself is not sufficient for uptake, but also the mobility of the mineral element is required which can be clearly seen in bacterial treatments there by higher presence of zinc levels in maize plants treated with PGPR. A “*Z*”-score statistical ranking method was followed to identify the promising bacterial isolate among all the strains and P29 strain was marked as the top strain compared to other bacteria for maize growth promotion.

## 5. Conclusion

Our study with PGPR and maize revealed that inoculation with beneficial rhizobacteria is an effective method for enhancing the growth of maize and maintaining the nutrient quality. Identified potential plant growth promoting rhizobacteria (P29) could be used as bioinput for improving the plant productivity as a substitute to chemical fertilizers and also to correct the nutrient deficiencies in maize for sustainable agriculture.

## Figures and Tables

**Table 1:** Zinc solubilizing ability of *Pseudomonas* and *Bacillus *isolates with insoluble zinc substrates on solid medium.

Bacterial isolates	Solubilization zone diameter (in mm)	
	ZnCO_3_	ZnO
P17	20	31
P21	21	24
P29	22	27
P33	22	27
P74	21	23
B40	22	31
B61	18	19
B114	14	17
B116	20	24
B118	20	26

**Table 2:** Biometric growth parameters of maize seeds treated with ZSBs and inorganic source of zinc.

Treatment	RV (cc)	SL (cm)	TDM (gm)	LA (cm^2^)
Control	9.8^j^ (±0.45)	78.8^h^ (±3.63)	9.16^h^ (±0.422)	627.7^i^ (±28.93)
ZnSO_4_	13.8^h^ (±0.64)	85.1^fg^ (±3.92)	15.25^a^ (±0.703)	1161.3^a^ (±53.52)
Priming	15.0^fg^ (±0.69)	96.0^c^ (±4.42)	12.87^b^ (±0.593)	861.0^f^ (±39.68)
B61	15.0^fg^ (±0.69)	97.8^b^ (±4.51)	11.36^d^ (±0.523)	908.3^e^ (±41.86)
B40	15.7^de^ (±0.72)	92.1^d^ (±4.24)	11.98^c^ (±0.552)	955.5^d^ (±44.04)
B116	16.7^c^ (±0.77)	110.1^a^ (±5.07)	12.78^b^ (±0.589)	1113.8^b^ (±51.33)
B114	16.2^cd^ (±0.75)	92.4^d^ (±4.26)	9.81^fg^ (±0.452)	901.7^e^ (±41.56)
B118	16.3^c^ (±0.76)	89.0^e^ (±4.10)	12.08^c^ (±0.557)	1041.8^c^ (±48.02)
P33	15.3^e–g^ (±0.71)	95.8^c^ (±4.42)	12.08^c^ (±0.557)	982.5^d^ (±45.28)
P29	18.3^b^ (±0.84)	84.7^fg^ (±3.90)	12.96^b^ (±0.597)	1147.5^ab^ (±58.02)
P74	14.8^g^ (±0.68)	75.5^i^ (±3.48)	10.13^f^ (±0.467)	851.7^fg^ (±39.25)
P17	9.8^j^ (±0.45)	73.5^i^ (±3.39)	7.38^i^ (±0.340)	611.8^i^ (±28.2)
P21	19.8^a^ (±0.91)	96.0^c^ (±4.43)	10.61^e^ (±0.489)	790.7^h^ (±36.44)
ZSB	12.8^i^ (±0.59)	86.3^f^ (±3.98)	9.67^g^ (±0.446)	859.7^f^ (±39.62)
FYM	15.5^ef^ (±0.71)	83.5^g^ (±3.85)	9.08^h^ (±0.418)	819.3^gh^ (±37.76)

LSD	0.57	2.0	0.42	35.5

Values in the parentheses are standard errors and parameters were recorded at 60 DAS.

Values superscribed by the same alphabet are not significantly different according to Fisher's least significant difference test (*P* < 0.05).

RV: root volume; SL: shoot length; TDM: total dry mass; LA: leaf area.

**Table 3:** Macronutrients uptake pattern of maize, seed treated with ZSBs, and inorganic source of zinc.

Treatment	Macronutrients (%)					
	Total “P”	Total “N”	K	Na	Ca	Na : K ratio
Control	0.18^f^ (±0.160)	1.428^j^ (±0.160)	1.6^c^ (±0.074)	0.004^d^ (±0.00018)	0.319^d^ (±0.015)	0.0025
ZnSO_4_	0.15^i^ (±0.217)	1.883^bc^ (±0.217)	1.8^b^ (±0.083)	0.005^c^ (±0.00023)	0.434^a^ (±0.020)	0.0028
Priming	0.19^e^ (±0.150)	1.568^fg^ (±0.150)	1.5^d^ (±0.069)	0.006^b^ (±0.00028)	0.299^g^ (±0.014)	0.004
B61	0.22^b^ (±0.170)	1.848^c^ (±0.170)	1.6^c^ (±0.074)	0.004^d ^(±0.00018)	0.339^c^ (±0.016)	0.0025
B40	0.28^a^ (±0.099)	1.638^de^ (±0.099)	1.8^b^ (±0.083)	0.002^f^ (±0.00009)	0.197^k^ (±0.009)	0.0011
B116	0.11^j^ (±0.114)	1.533^gh^ (±0.114)	1.4^e^ (±0.065)	0.003^e^ (±0.00014)	0.227^j^ (±0.010)	0.0021
B114	0.16^h^ (±0.149)	1.883^bc^ (±0.149)	1.3^f^ (±0.060)	0.001^g^ (±0.00005)	0.298^g^ (±0.014)	0.0008
B118	0.15^i^ (±0.156)	1.673^d^ (±0.156)	1.4^e^ (±0.065)	0.004^d^ (±0.00018)	0.311^e^ (±0.014)	0.0029
P33	0.18^f^ (±0.171)	1.603^ef^ (±0.171)	1.5^d^ (±0.069)	0.005^c^ (±0.00023)	0.341^c^ (±0.016)	0.0033
P29	0.20^d^ (±0.052)	2.268^a^ (±0.052)	2.0^a^ (±0.092)	0.005^c^ (±0.00023)	0.104^l^ (±0.005)	0.0025
P74	0.17^g^ (±0.177)	1.603^ef^ (±0.177)	1.5^d^ (±0.069)	0.003^e^ (±0.00014)	0.354^b^ (±0.016)	0.002
P17	0.21^c^ (±0.169)	1.498^hi^ (±0.169)	1.3^f^ (±0.060)	0.002^f^ (±0.00009)	0.338^c^ (±0.016)	0.0015
P21	0.17^g^ (±0.143)	1.673^d^ (±0.143)	1.4^e^ (±0.065)	0.003^e^ (±0.00014)	0.285^h^ (±0.013)	0.0021
ZSB	0.16^h^ (±0.152)	1.463^ij^ (±0.152)	1.5^d^ (±0.069)	0.007^a^ (±0.00032)	0.303^f^ (±0.014)	0.0047
FYM	0.18^f^ (±0.133)	1.568^fg^ (±0.133)	1.3^f^ (±0.060)	0.002^f^ (±0.00009)	0.266^i^ (±0.012)	0.0015

LSD	0.015	0.046	0.042	0.00012	0.002	

Values in the brackets are standard errors and parameters were recorded at 60 DAS.

Values in the columns are means of two independent experiments with six replicates each time.

**Table 4:** Micronutrients uptake pattern of maize, seed treated with ZSBs, and inorganic source of zinc.

Treatment	Micronutrients (ppm)				
	Zn	Mg	Fe	Cu	Mn
Control	158.6^i^ (±7.3)	0.16^cd^ (±0.0074)	481.4^c^ (±22)	2.9^c^ (±0.13)	34^h^ (±1.6)
ZnSO_4_	188.6^g^ (±8.7)	0.23^a^ (±0.0106)	626.7^b^ (±29)	4.8^a^ (±0.22)	60^a^ (±2.8)
Priming	178.6^h^ (±8.2)	0.15^de^ (±0.0069)	488.4^c^ (±23)	3.0^c^ (±0.14)	34^h^ (±1.6)
B61	243.4^c^ (±11.2)	0.17^bc^ (±0.0078)	672.8^a^ (±31)	2.9^c^ (±0.13)	51^b^ (±2.4)
B40	192^g^ (±8.8)	0.11^h^ (±0.0051)	226.4^f^ (±10)	3.7^b^ (±0.17)	46^c^ (±2.1)
B116	185.2^gh^ (±8.5)	0.11^h^ (±0.0051)	234.8^f^ (±11)	1.5^e^ (±0.07)	37^fg^ (±1.7)
B114	235.2^d^ (±10.8)	0.14^ef^ (±0.0065)	367^d^ (±17)	2.1^e^ (±0.10)	40^e^ (±1.8)
B118	231.8^de^ (±10.7)	0.15^de^ (±0.0069)	282.7^e^ (±13)	1.5^e^ (±0.07)	24^i^ (±1.1)
P33	225^e^ (±10.4)	0.18^b^ (±0.0083)	381^d^ (±18)	0.9^f^ (±0.04)	38^ef^ (±1.8)
P29	278.8^a^ (±12.8)	0.04^i^ (±0.0018)	155.2^g^ (±7)	3.1^c^ (±0.14)	60^a^ (±2.8)
P74	230^de^ (±10.6)	0.16^cd^ (±0.0074)	495^c^ (±23)	1.9^d^ (±0.09)	44^cd^ (±2.0)
P17	259^b^ (±11.9)	0.16^cd^ (±0.0074)	381^d^ (±18)	0.9^f^ (±0.04)	35^gh^ (±1.6)
P21	232^de^ (±10.7)	0.14^ef^ (±0.0065)	707^a^ (±33)	0.5^g^ (±0.02)	25^i^ (±1.2)
ZSB	192^g^ (±8.8)	0.13^fg^ (±0.0060)	594^b^ (±27)	0.7^fg^ (±0.03)	43^d^ (±2.0)
FYM	212^f^ (±9.8)	0.13^fg^ (±0.0060)	359^d^ (±17)	0.7^fg^ (±0.03)	21^j^ (±1.0)

LSD	7	0.011	36.2	0.27	2.5

Values in the brackets are standard errors and parameters were recorded at 60 DAS.

Values superscribed by the same alphabet are not significantly different according to Fisher's least significant difference test (*P* < 0.05).
